# Establishing Bedding Requirements on Trailers Transporting Market Weight Pigs in Warm Weather

**DOI:** 10.3390/ani4030476

**Published:** 2014-07-25

**Authors:** Rebecca Kephart, Anna Johnson, Avi Sapkota, Kenneth Stalder, John McGlone

**Affiliations:** 1 Department of Animal Science, Iowa State University, Ames, IA 50011, USA; E-Mails: rkdavis@iastate.edu (R.K.); stalder@iastate.edu (K.S.); 2 Laboratory of Animal Behavior, Physiology and Welfare, Department of Animal and Food Sciences, Texas Tech University, Lubbock, TX 79409, USA; E-Mails: asapkota@purdue.edu (A.S.); john.mcglone@ttu.edu (J.M.)

**Keywords:** bedding, market-weight pig, transport losses, well-being

## Abstract

**Simple Summary:**

Transport is an inevitable process in the modern swine industry. Trailers transporting pigs are bedded with straw, wood shavings, corn stover, or sand. Excess bedding may detrimentally affect the micro-environment inside the trailer during warm weather and in turn negatively affect animal based measures and transport losses. These experiments aim to determine the amount of bedding that is ideal for market weight pig transport during warm weather.

**Abstract:**

During warm weather, incorrect bedding levels on a trailer transporting market weight pigs may result in heat stress, fatigue, and death. Two experiments were conducted in June and July of 2011; Experiment 1 used 80 loads (*n =* 13,887 pigs) to determine the effects of two bedding levels (3 (68.1 kg) or 6 bags (136.2 kg) of wood shavings/trailer [each bag contained 22.7 kg, 0.2 m^3^]) on pig measures (surface temperature, vocalizations, slips and falls, and stress signs). Experiment 2 used 131 loads (*n =* 22,917 pigs) to determine the effects of bedding (3 *vs*. 6 bags) on transport losses (dead, sum of dead- and euthanized- on arrival; non-ambulatory, sum of fatigued and injured; total transport losses sum of dead and non-ambulatory). Bedding did not affect surface temperature, vocalizations, or slips and falls (*p* = 0.58, *p* = 0.50, and *p* = 0.28, respectively). However, pigs transported on 6 bags/trailer had 1.5% more stress signs than pigs transported on 3 bags/trailer (*p* < 0.01). No differences were observed between bedding levels for non-ambulatory, dead, or total transport losses (*p* = 0.10, *p* = 0.67, and *p* = 0.34, respectively). Within the context of these experiments, bedding level did not result in deleterious effects on pig measures or transport losses. However, using more bedding may result in higher costs to the industry. Therefore, 3 bags of bedding/trailer may be used when transporting market weight pigs during warm weather in the Midwestern U.S.

## 1. Introduction

In 2011, ~110 million pigs were marketed in the U.S. [1]. Transporting pigs is essential to multi-site pork production. For pigs, the marketing process is a combination of potentially novel (defined as the first exposure), unfamiliar (defined as infrequent exposures), and physically exerting experiences that could be perceived as stressful [2]. If the pig is unable to cope with these stressors, increased transport losses and decreased meat quality may result [3,4,5]. The term “*transport losses*” refers to pigs that become non-ambulatory (pigs that are unable to keep up with the group and may be injured) or are classified as dead on arrival at the plant [2].

The conditions under which pigs are handled and transported can have a direct impact on their well-being, which may result in increased transport losses. In the U.S., pigs are transported in trailers which rely on passive ventilation where air flow is dependent upon thermal buoyancy, movement of the vehicle, and wind speed [6,7]. An observational study by Haley *et al*. (2008) noted that as temperatures increased from 26 to 30 °C, in-transit losses increased approximately two-fold [8]. Another study by Dewey *et al*. (2009) reported that as the environmental temperature increased by 1 °C, the internal truck temperature increased by 0.99 °C. As the environmental humidity increased by 1%, the internal; truck temperature increased by 0.11 °C. In addition, as the 90th percentile of temperature increased by 1 °C, the in-transit loss increased 1.26 times [9]. To control the trailer environment, truckers provide bedding to help absorb urine and fecal matter, reduce slips and falls, and maintain the pigs’ thermo-neutral zone [6,7,8]. The U.S. industry’s Transport Quality Assurance (TQA) program defines appropriate bedding as straw, corn stover, wood shavings, or sand and provides recommendations for bedding levels. However, these recommendations are based on experiential information rather than scientific data [7]. Therefore, the objectives of these experiments were to compare the effects of 2 bedding levels on the (1) pig measures at the time of unloading and (2) transport losses during warm weather for market weight pigs.

## 2. Materials and Methods

### 2.1. General Procedures

#### 2.1.1. Treatments and Experimental Design

Both experiments compared 2 bedding levels on trailers transporting market weight pigs: 3 (68.1 kg) and 6 (168.1 kg) bags (0.2 m^3^; 22.7 kg) of wood shavings/trailer. One treatment was randomly assigned to each trailer by the trucking companies. The data was collected over two 1-wk periods during June and July 2011.

#### 2.1.2. Animals, Farms, and Pig Handling

The protocol for these experiments was approved by the Iowa State University Institutional Animal Care and Use Committee. The company’s loading crew identified market weight barrows and gilts (PIC) and moved them from the home pens to the loading ramp entrance. The trucker moved these pigs up the loading ramp and onto the trailer. During loading, the loading crew and the trucker used a combination of sort boards, rattles, paddles, and electric prods (the number of times these devices were used was not recorded). On average, 7 pigs were moved as a group from their home pens to the trailer as recommended by the TQA program. The pigs were transported from commercial finishing facilities to a commercial processing plant. All finishing facilities and the processing plant were located in Iowa. Transport occurred throughout the day and night. Upon arrival at the plant, the trucker unloaded the pigs from the truck, and plant personnel moved the pigs from the bottom of the unloading ramp to the lariage pens. During unloading, plant personnel and the trucker used a combination of paddles, rattles, and boards.

#### 2.1.3. Transport Trailers and Trailer Stocking Density

All pigs were transported on aluminum drop deck (pot belly) trailers 17 m in length with diamond plate flooring. These were owned and operated by the trucking companies contracted through the plant. All compartments in the trailer were stocked according to the industry’s current standard operating procedure of 0.41 m^2^/pig or ~173 pigs/load. The plant provided data on the number of pigs/trailer and the average weight of pigs on a trailer. For these experiments a trailer stocking density value was calculated and added to the statistical model because previous work has found trailer stocking density is an important variable in affecting animal based measures and transport losses [10,11,12,13].

Trailer stocking density = (average pig weight per trailer) × (pigs per trailer)/(floor space in trailer)

#### 2.1.4. Environmental Measures at Loading and Unloading

At loading, relative humidity and ambient air temperature (temperature) were measured either with a mini thermo-anemometer with humidity (*n =* 77; model 45158, Extech Instruments Nashua, NH, USA; accurate ± 0.4% for relative humidity and ±1 °C for temperature) or by a weather station closest to the farm (*n =* 54; ≤32.8 km from the farm). The Citizen Weather Observer Program (CWOP) dictates these weather stations are accurate to ±1.1 °C [14]. The National Oceanic and Atmospheric Association (NOAA) which oversees CWOP, only uses dew point for accuracy. Therefore, CWOP dictates dew point should be accurate to ±2.2 °C. During loading, temperature and relative humidity ranged from 10.6 to 38.3 °C and 33.2% to 98.0%, respectively.

During unloading, temperature and dew point were measured at an airport 16.9 km from the plant (1088 hygrothermometer Technical Service Laboratory Fort Walton Beach, FL, USA). Relative humidity was then calculated from dew point and temperature measurements by the outputting computer (accurate ± 0.003 °C). Temperature and relative humidity during unloading ranged from 16.1 to 43.4 °C and 43.0% to 97.4%, respectively. Temperature (T) and relative humidity (RH) were used to calculate a Temperature Humidity Index (THI) using the following equation provided by the NOAA [15] and was included in the statistical model [10]:

THI = T − {[0.55 − (0.0055 × RH_decimal_)](T − 14.5)}

### 2.2. Experiment 1: Effects of Trailer Bedding Levels on Market Weight Pig Measures and Bedding Moisture during Warm Weather

This experiment used 80 loads; 48 loads had 3 bags/trailer and 32 loads had 6 bags/trailer. Some trailers were used multiple times prior to being thoroughly cleaned and disinfected.

#### 2.2.1. Pig Measures

Vocalizations, slips and falls, and stress signs were collected on a random sample of pigs at unloading using live observation. A random sample was defined as ignoring ~10 pigs at the beginning of unloading, counting measures for 50 pigs (group A), ignoring a further ~10 pigs, and counting measures for another 50 pigs (group B). This provided 100 pigs/load. Vocalizations were defined as an extended sound of high amplitude and frequency produced with an open mouth [2]. Slips were defined as a knee or hock touching the ground; falls were defined as a pig’s body touching the ground [6]. Slips and falls were tallied as a single measure. Stress signs were defined as open mouth breathing, muscle tremors, and red-blotchy skin [16,17]. Surface temperature was measured on 5 random pigs in group A and 5 random pigs in group B (total of 10 pigs/load) laterally near the midline. Surface temperature was measured with a dual laser infrared thermometer laterally near the midline (model 42570, Extech Instruments Nashua, NH, USA; accurate ± 1 °C).

#### 2.2.2. Transport Events

Transport events in this experiment were loading, transport, wait time, and unloading. Loading was defined as the time interval from the first pig’s first foot stepping onto the trailer until the last pig’s last foot stepped onto the trailer. Transport was defined as the time interval from when the last pig’s last foot stepped onto the trailer until the trailer arrived at the plant. Wait time was defined as the time interval from when the trailer arrived at the plant until the first pig’s first foot stepped off the truck. Unloading was defined as the time interval from when the first pig’s first foot stepped off the truck until the last pig’s last foot stepped off the truck. Total transport time was the time from when the first pig’s first foot stepped onto the trailer (start of loading) until the last pig’s last foot stepped off the trailer (end of unloading).

#### 2.2.3. Bedding Moisture

There were 77 bedding samples taken from trailers with 3 bags/trailer: 0 loads, *n =* 13; 1 load, *n =* 20; 2 loads, *n =* 15; 3 loads, *n =* 9, and ≥4, *n =* 20. There were 41 samples taken from trailers with 6 bags/trailer: 0 loads, *n =* 6; 1 load, *n =* 8; 2 loads, *n =* 12; 3 loads, *n =* 5; ≥4 loads, *n =* 10. Fresh samples (0 loads) were defined as bedding that had not been previously used for transporting pigs. A fresh bedding sample of ~45 g was collected. After each trailer had unloaded at the plant, a used bedding sample, defined as bedding which had transported ≥1 trailer loads of pigs was collected. Half of the used bedding was collected from the bottom trailer deck and the remainder was collected from the top deck. Each used bedding sample collected was ~410 g. Bedding samples were stored at room temperature (~21 °C) for no longer than 1 wk after trial completion.

Bedding moisture was determined following a standard operating procedure for drying samples. A tin measuring 7.6 cm wide by 2.2 cm deep (model A90, Wilkinson Industries Inc., Fort Calhoun, NE, USA) was weighed. Each bedding sample was kneaded by hand inside the closed storage bag for ~30 s. Two, 3 to 6 g subsamples (subsample A and B) were removed from the bag using a spoon. Subsample A was placed in one tin and subsample B was placed in a second. The bedding subsample in its respective tin was weighed (accurate ± 0.03 mg; model AT261 DeltaRange, Metler-Toledo GmBh Laboratory and Weighing Technologies, Greifensee, Switzerland) to determine wet weight. Bedding subsamples were dried for ~20 to 24 h at 100 °C in a convection oven (model DKN810, Yamato Scientific America Inc., Santa Clara, CA, USA). After drying, subsamples were re-weighed; this was defined as the dry weight. Moisture percent for each subsample was calculated using the following equation [15]:

Moisture percent = [(dry weight)/(wet weight)] × 100

A standard deviation of moisture percentage between subsample A and subsample B and an average of the moisture percent of subsample A and subsample B were calculated. Between subsample A and subsample B, the coefficient of variation (CV) was calculated using the following equation:

CV = (Standard deviation/average) × 100

If the CV ≥ 10 the sample was re-subsampled and dried a second time (*n =* 14). If the sample was still found to be too variable on the second drying, that sample was removed from the data set (*n =* 0). The data from bedding moisture will be presented descriptively separated by the number of loads on the bedding, ranging from 0 to ≥4 loads.

### 2.3. Experiment 2: Effects of Trailer Bedding Levels on Market Weight Pig Transport Losses during Warm Weather

This experiment used 131 loads; 88 loads had 3 bags/trailer and 43 loads had 6 bags/trailer.

#### Transport Losses at the Plant

Processing facility employees identified dead (sum of euthanized- and dead on arrival) and non-ambulatory pigs (sum of fatigued and injured) [2]. Total transport losses were defined as the summation of dead and non-ambulatory pigs.

### 2.4. Statistical Analysis

For both experiments, data were evaluated for missing and erroneous values by using the filter feature in Excel (Microsoft Office 2010, Microsoft Redmond, WA, USA). The remaining analyses were completed using SAS software (SAS V 9.2 Institute Inc., Cary, NC, USA). Using the means and sort procedures data was checked for erroneous and potential outlier data points. Data that was identified as a potential outlier was checked against the original data. If correct it was simply highlighted in the excel data, if incorrect that value was substituted per the original data. A *p*-value ≤ 0.05 was considered significant for both experiments. A *p*-value ≤ 0.10 was considered trending for both experiments. Each variable collected was evaluated on whether it should be present in the model (Table 1). Those variables that were dictated by previous research to affect the response were retained for the final model as well as any other variables found to be significant during model development.

**Table 1:** Variables that might have affected the response were attempted in the models.

Variable	*p*-Value
Bedding moisture	*p* = 0.24
Day	*p* = 0.61
Loading time	*p =* 0.92
Previous trips taken by the trailer	*p* = 0.96
Researcher	*p =* 0.19
Sex of pig	*p =* 0.08
Truck type	*p* = 0.65
Unloading time	*p =* 0.80
Wait time at the plant	*p* = 0.95

#### 2.4.1. Experiment 1. Effects of Trailer Bedding Levels on Market Weight Pig Measures and Bedding Moisture during Warm Weather

Because researchers sometimes counted more or less than, 50 pigs/group, data for vocalizations, slips and falls, and stress signs were analyzed as a percent of the pigs counted:

Percent pig measure = [(number of times a measure was counted)/(number pigs counted in that group)] × 100

Furthermore, the SAS program was used to create a new variable from the percent of vocalizations, slips and falls, and stress signs from group A and group B of 50 (e.g., [percent stress signs group A + percent stress signs group B]/2). Surface temperature was analyzed as an average of the 5 pigs measured/group (10 pigs measured/load).

The independent variables including wait time, number of previous loads and the respective interactions were removed from the final analysis model were not significant sources of variation for the present data. Additionally, the independent variable total transport time was confounded with farm and hence both could not be included in any statistical analysis. Finally, the independent variable transport day effectively was included as part of the THI because the THI for each day pigs were transported was included in the final statistical model. Therefore, data were analyzed using a mixed model where the response variables, surface temperature, vocalizations, slips and falls, and stress signs, were analyzed using bedding level as a fixed effect, THI at unloading and trailer stocking density as linear covariates, and farm as a random effect.

#### 2.4.2. Experiment 2: Effects of Trailer Bedding Levels on Market Weight Pig Transport Losses during Warm Weather

Analysis of non-ambulatory, dead, and total transport losses per trailer was performed using a generalized mixed model. The data approximated a Poisson distribution and was log transformed by the GLIMMIX procedure prior to statistical analysis. The model used bedding level as a fixed effect, THI at unloading and trailer stocking density as linear covariates, and farm as a random effect. The ILINK was used to back-transform least squares means into their original unit of measure for ease of interpretation.

## 3. Results and Discussion

### 3.1. Experiment 1: Effects of Trailer Bedding Levels on Market Weight Pig Measures and Bedding Moisture during Warm Weather

#### 3.1.1. Pig Measures

No differences were observed between 3 and 6 bags/trailer for surface temperature, vocalizations, or slips and falls indicating that the use of straw bedding at these temperatures (*p* ≥ 0.28; Table 2). The TQA program defines appropriate bedding as straw, corn stover, wood shavings, or sand [7]. However, other sources [18,19,20] suggest straw bedding may be too warm when temperatures exceed 15.6 °C because it may insulate the trailer. However, in these studies, the internal microclimate of the trailer did not detrimentally affect pig surface temperature. Bedding has multiple uses when transporting pigs. One use is to reduce slips and falls and perhaps overall stress experienced by the animal [21]. Although, not different in this study, slips and falls were collected after the pigs stepped off the trailer and away from the bedding source. Thus, it cannot be concluded that more bedding did not aid in reducing the number of slips and falls experienced by the pig during transit or while still on the trailer during unloading. Studies collecting slips and falls during transit on the trailer should be conducted. Finally, vocalizations are a non-invasive measure which may indicate distress [22,23,24]. Kiley [21] has described 13 different types of pig vocalizations being expressed at different ages and within a variety of situations, for example social-greeting or non-social-startle. Furthermore, studies have found that squeal type vocalizations are associated with unpleasant situations [22,23,25]. Although vocalizations were not different in the context of this study between bedding levels, they may still be a useful non-invasive measure when assessing how pigs are coping with the transport process.

Stress signs at the time of unloading were 1.5% higher when pigs were transported using 6 instead of 3 bags/trailer (*p* < 0.01; Table 2). It is unclear as to why stress signs at unloading were higher in 3 *vs*. 6 bags. One hypothesis is that pigs on the trailer during transport struggled to maintain footing resulting in stress signs being higher at unloading. However, this theory would need to be considered in future studies, by placing cameras inside the trailer and monitoring each pig throughout transportation. In addition, even though stress signs were different, they were very low in this study.

**Table 2:** Experiment 1. Bedding level by pig measure LSMeans (±SE) measured ^1^ during unloading for market weight pigs ^2^.

	Bags of Bedding ^3^			
Pig Measure	3 *n =* 48	6 *n =* 32	*p-*Value	R^2^
Surface temperature, °C	32.9 ± 0.3	33.1 ± 0.3	0.58	0.47
Vocalizations	1.8 ± 0.4	2.2 ± 0.5	0.50	0.10
Slips and falls, % of pigs counted	2.2 ± 0.8	3.0 ± 0.8	0.28	0.13
Stress signs, % of pigs counted	0.1 ± 0.3	1.6 ± 0.4	<0.01	0.20

^1^ Pig measures were surface temperature (ST), vocalizations, slips and falls, and stress signs. ST was measure on 10 pigs/load with a dual laser infrared thermometer laterally midline. Vocalizations, slips and falls, and stress signs were tallied for 100 pigs/load; ^2^ Based on 80 trailer loads of market weight pigs; ^3^ 0.2 m^2^ bags of wood shavings.

#### 3.1.2. Transport Events

The mean loading (35 min) and unloading (16 min) times for the current study is similar to previous studies of 38 min [17], 45 min [26] and 18 min [17] respectively (Table 3). The mean transport time in the current study (138 min) was more than double compared to previous studies at 59 min [17] and at 107.1 min [27]. A possible explanation for increased transport time in this study was the distance between farms and plant. The shortest distance from farm to plant was 23.2 km and the furthest was 284.9 km, with an average distance of 189.4 km. Gesing *et al*. noted the finishing sites used in their study were only 85 km [17,28]. The wait time observed in the current study (20 min) was longer than that reported by Gesing *et al*. (9 min) [17]. However, Pilcher reported mean wait time of 21 min [26] and Gesing and others in 2010 reported a mean wait time of 22 min [27]. Wait time can be affected by a variety of factors such as time of arrival, time of trucks ahead to unload and labor availability at the plant.

**Table 3:** Experiment 1. Descriptive statistics for transport events ^1^ for market weight pigs ^2^.

Event, Min	Mean	SD ^3^	Min ^4^	Max ^5^
Loading	35	12	15	84
Transport	158	40	32	222
Wait time	20	13	2	66
Unloading	16	5	3	28
Total Time	228	44	77	298

^1^ Transport events were loading, transport, wait time, unloading, and total time. Loading was the time from when the first pig stepped on to the trailer until the last pig stepped onto the trailer. Transport was as the time from when the last compartment on the trailer was closed until the truck arrived at the plant. Wait time was defined as the time from when the truck arrived at the plant until the first pig stepped off. Unloading was as the time from the first pig stepped off the trailer until the last pig stepped off the trailer the trailer. Total time is the time from when the first pig steps onto the trailer until the last pig steps off the trailer; ^2^ Based on 77 trailers of pigs; ^3^ SD abbreviation for standard deviation; ^4^ Min abbreviation for minimum; ^5^ Max abbreviation for maximum.

Transportation event times will need to be carefully monitored by trucking companies, processing facilities, and the truckers due to changes made by the U.S. Department of Transportation (DOT). As of 1 July 2013, the DOT hours-of-service safety regulation states that after 8 h of driving the trucker must take a 30 min break away from the truck [29]. For transportation of non-animal related goods this will likely not be a challenge. However, if live animals are being transported several challenges to their well-being are recognized including a build-up of heat and humidity in the warm months [9,30].

#### 3.1.3. Bedding Moisture

Fresh bedding (0 loads) had ~9% moisture. After one load, 6 bags/trailer resulted in 14% less moisture than 3 bags/trailer. However, as number of loads increased, more bedding did not provide additional moisture absorption, with subsequent loads having approximately 69% moisture (Table 4).

**Table 4:** Experiment 1. Descriptive statistics for bedding moisture (%) between 3 and 6 bags of bedding/trailer transporting market weight pigs in warm weather ^1^.

Loads ^6^	Bedding Levels (Bags/Trailer) ^2^							
	3				6			
	Mean	SD ^3^	Min ^4^	Max ^5^	Mean	SD	Min	Max
0	8.5	2.1	5.3	13.1	9.3	4.4	6.3	17.9
1	69.1	8.1	55.4	87.6	55.6	11.9	47.1	76.2
2	71.0	6.9	57.1	81.7	72.5	7.3	61.3	85.6
3	69.1	3.3	64.1	74.4	67.3	5.2	62.5	73.3
≥4	70.0	9.9	50.1	85.9	64.1	12.1	42.3	81.8

^1^ There were 77 bedding samples taken from trailers with 3 bags/trailer: 0 loads, *n =* 13; 1 load, *n =* 20; ^2^ loads, *n =* 15; 3 loads, *n =* 9, and ≥4, *n =* 20. There were 41 samples taken from trailers with 6 bags/trailer: 0 loads, *n =* 6; 1 load, *n =* 8; 2 loads, *n =* 12; 3 loads, *n =* 5; ≥4 loads, *n =* 10; ^2^ ~0.2 m^3^ bags of wood shavings/trailer; ^3^ SD abbreviation for standard deviation; ^4^ Min abbreviation for minimum; ^5^ Max abbreviation for maximum; ^6^ Zero loads indicate samples were bedding not previously used being placed onto the clean trailer floor prior to loading. One load or more indicates those samples which have been on the trailer when pigs were transported from farm to plant.

Lack of increasing moisture with subsequent loads suggests that only fresh wood shaving bedding is effective at absorbing pig waste. Wood shavings have been reported to be less absorbent than straw or corn stover (1.15 *vs*. 1.97 *vs*. 2.70 mean absorbency factor, respectively [31,32]. Data in the current study supports the TQA guidelines, suggesting trailers should be washed out and fresh bedding applied after every load [3,7]. Although bacterial load in the bedding does not directly threaten the pigs being transported to a packing plant, bedding could fall out of the trailer raising a potential biosecurity concern and it could be a threat if the truck goes to another pig or livestock operation [33]. Biosecurity has sharply come into focus in the U.S. due to the outbreak of Porcine Epidemic Diarrhea virus (PEDv). Although multiple trips were taken before trailers were washed and fresh bedding applied in these studies, these management practices are currently being reassessed. Finally, fear pheromones released in pig urine may increase stress for the pigs currently being loaded and transported [34,35,36]. Further studies on bedding type, bacterial load counts and the application of appeasing pheromones with their subsequent effects on the animal should be conducted.

Individual company protocols vary in the frequency of complete trailer washout, and application of fresh bedding. Such differences reflect numerous considerations based on cost, effects to the environment, and animal well-being. For example, trailer wash out can range from $15 to $190 [37]. Washing out trailers between each load and re-bedding the trailer has been estimated to cost ~$8 million and $108 million annually [38]. However, this estimate does not include potential lost income to the driver while washing the trailer or environmental implications for water usage and bedding disposal. In addition, using 3 instead of 6 bags in warm weather (defined as temperature ranging 16.1 °C to 43.4 °C) has been estimated to save $13 million [38]. Hence, adding the cost of washout, Kephart and others [38] found that using 3 bags/trailer and washing out after every load would cost between ~$22 million and $121 million annually. A cost benefit analysis for using fresh bedding after every load, in relation to overall swine well-being improvements is suggested.

#### 3.1.4. Temperature Humidity Index at Unloading

It was observed that as THI decreased from ~20 to 15, slips and falls at the time of unloading tended to increase ~22% (*p* = 0.09; R^2^ = 0.13; data not presented). One possible explanation for this is that pigs are more active when THI is closer to their thermo-neutral zone and therefore move off the trailer more quickly thus creating the potential for more slips and falls.

**Figure 1:**
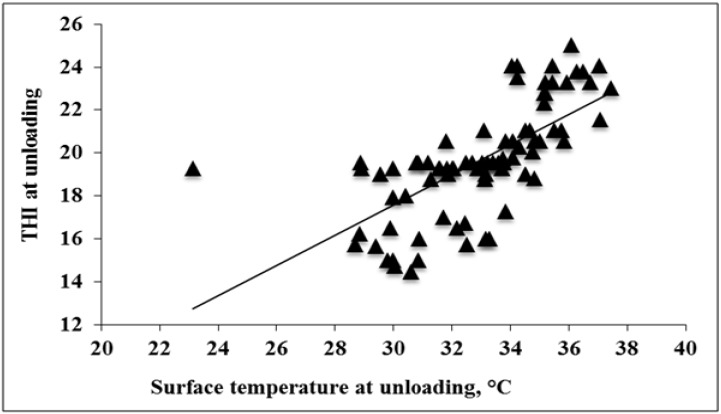
Experiment 1. Effects of temperature humidity index (THI) at unloading on surface temperature of pigs at unloading (*p* < 0.01; R^2^ = 0.47).

**Figure 2:**
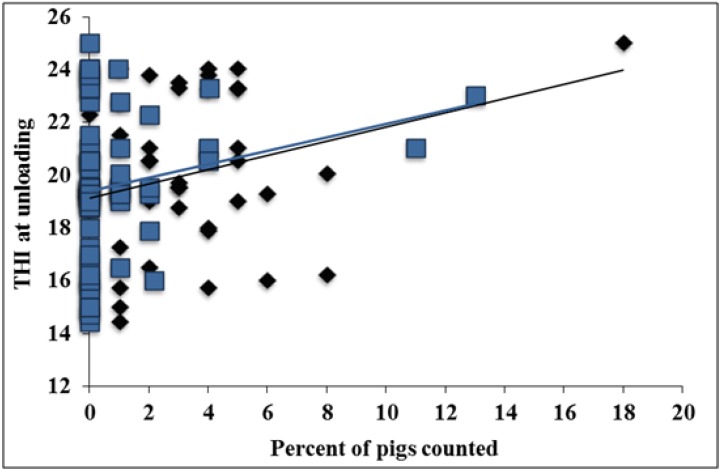
Experiment 1. Effects of temperature humidity index (THI) at unloading on vocalizations (◆) and stress signs (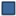) at unloading including linear trend lines (respectively; *p* < 0.01, R^2^ = 0.10; *p* = 0.04, R^2^ = 0.20).

As THI increased from ~13 to 23, surface temperature increased ~14 °C (*p* < 0.01; Figure 1). As THI increased from ~19 to 24 vocalizations increased ~18% and stress signs increased ~13% (*p* < 0.01 and *p* = 0.04, respectively; Figure 2). The relationship between THI and surface temperature were moderate (R^2^ = 0.47). The relationship between THI- and vocalizations and stress signs was weak (R^2^ = 0.10 and R^2^ = 0.20, respectively). A pig’s thermo-neutral zone ranges from 10 to 21 °C [39] and their normal core temperature ranges from ~39 to 40 °C [40]. Although surface temperature has been shown to be reflective of core temperature it is slower to reflect changes in core temperature than rectal measurement [41]. When pigs become heat stressed they will pant and increase blood flow to skin and limbs [42,43]. Increasing blood flow to the skin can cause discolored skin. It follows that this could also cause increased skin temperature. Although surface temperature ranges seen in this study are not reflective of heat stressed pigs, this may simply mean that the pigs’ physiological mechanisms for coping with heat were acting effectively.

#### 3.1.5. Trailer Stocking Density

In the current experiment, there was no observed trailer stocking density effects on pig surface temperature, vocalizations, or slips and falls (*p* = 0.31, R^2^ = 0.47; *p* = 0.19, R^2^ = 0.10; and *p* = 0.55, R^2^ = 0.13, data not presented). Direct comparisons for changes in pig surface temperature based on trailer stocking density have not been published. Ritter and others found that trailer stocking density did not affect rectal temperature between 0.39 and 0.49 m^2^/pig (~333 kg/m^2^ and 265 kg/m^2^ respectively) [12] and Chung and others [41] noted that as rectal temperature increased surface temperature increased in a linear manner.

**Figure 3:**
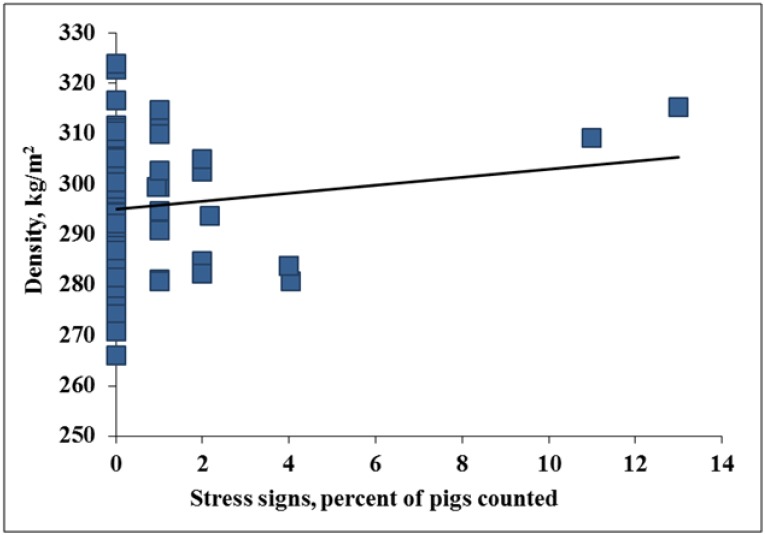
Experiment 1. Effects of trailer stocking density on stress signs (*p* = 0.03; R^2^ = 0.20).

However, as trailer stocking density increased in the current work from ~295 to 305 kg/m^2^ stress signs increased ~13% (*p* = 0.03, Figure 3). Pigs in the current study were transported at an average trailer stocking density of 296 m^2^/pig, but the trailer stocking density equation used factored in weight and number of pigs on the trailer and was presented as a continuous variable. This may be why the stress results in the current work disagree with Ritter and others [11] who reported that pigs transported at 0.52 m^2^/pig (~252 kg/m^2^) had a higher incidence of skin discoloration than pigs transported at 0.39, 0.42, or 0.46 m^2^/pig (~336, 312, and 285 kg/m^2^ respectively). This raises an interesting statistical discussion in regards to fixed effects and covariates, the use of both trailer stocking density and THI equations and in turn results, making comparison of these data sets challenging. Fixed effects allow comparison of discrete categories, but, unlike continuous effects, it is not possible to determine what is happening in the space between categories. Even with more or heavier pigs on the trailer, slips and falls may not increase if pigs are not rushed off the trailer and the floor is dry [21].

### 3.2. Experiment 2: Effects of Trailer Bedding Levels on Market Weight Pig Transport Losses during Warm Weather

#### 3.2.1. Bedding

No differences were observed between bedding levels for non-ambulatory, dead, or total transport losses (*p* ≥ 0.10; Table 5). The current study observed ~0.05 non-ambulatory pigs/load. Although non-ambulatory pigs are not tracked by the Food Safety and Inspection Service, Ritter *et al*. [12] used 23 previous studies and estimated that in 2006 non-ambulatory pigs occurred at a rate of 0.44% (~0.74 pigs/trailer). This study observed ~0.13 dead pigs/load. During 2011 in the U.S. ~0.26 dead pigs/load were observed [39]. Fitzgerald [10] found transport losses during summer were mainly dead pigs, which is reflected in the current study’s results (Table 5). Kephart and others [38] determined that in 2011 each dead pig cost the industry $178, therefore, dead pigs cost the U.S. swine industry ~$29 million.

**Table 5:** Experiment 1. Effects of bedding level on trailers transporting market weight pigs on transport losses ^1^.

Transport Losses, Pigs per Trailer	Bags of Bedding ^2^			
	3 *n =* 88	6 *n =* 43	*p-*Value	R^2^
NA	0.02 ± 0.02	0.09 ± 0.05	0.10	0.03
Dead	0.11 ± 0.04	0.13 ± 0.06	0.67	0.07
TTL	0.14 ± 0.04	0.22 ± 0.07	0.24	0.08

^1^ Processing facility employees identified dead (sum of euthanized- and dead on arrival) and non-ambulatory pigs (sum of fatigued and injured). Total transport losses were defined as the summation of dead and non-ambulatory pigs; ^2^ Based on 131 trailer loads of market weight pigs; ^3^ 0.2 m^2^ bags of wood shavings.

In the current study using wood shavings at 3 or 6 bags/trailer pig well-being was not detrimentally affected. The current TQA program recommends 2 bags of bedding when temperature >4.4 °C. The current study chose to compare 3 and 6 bags/trailer because it was determined that 3 bags/trailer barely covers the trailer floor. However, future work should compare 1, 2 and 3 bags/trailer over the warm months to decide if less bedding still offers acceptable pig well-being during transit.

#### 3.2.2. Temperature Humidity Index at Loading and Trailer Stocking Density

**Figure 4:**
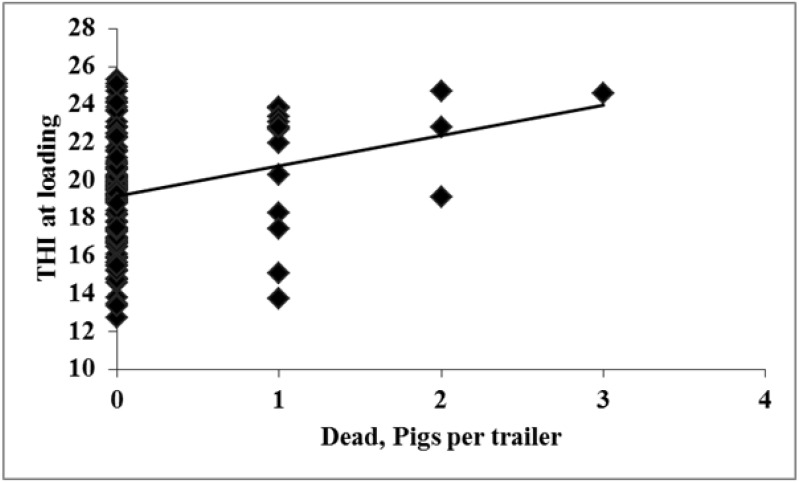
Experiment 2. Effects of THI at loading on dead (*p* = 0.01; R^2^ = 0.07).

**Figure 5:**
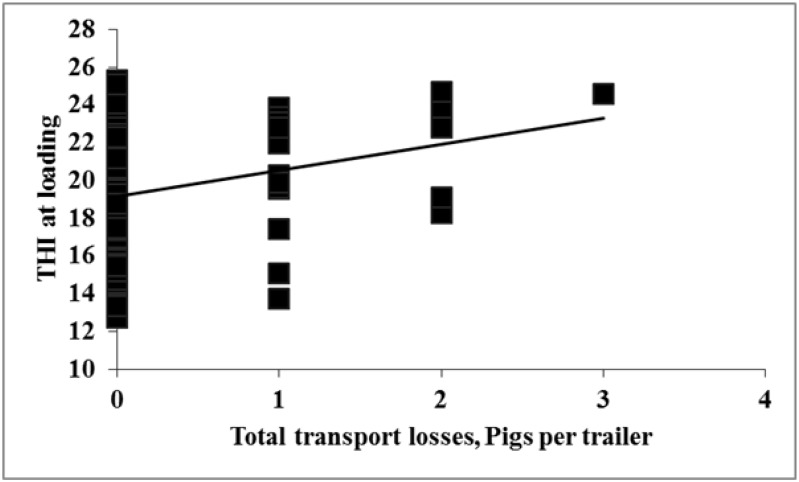
Experiment 2. Effects of THI at loading on total transport losses (*p* = 0.01; R^2^ = 0.08).

No effects were observed for THI at loading and of the number of non-ambulatory recorded at the plant (*p* = 0.51, R^2^ = 0.03; data not presented). However, it was observed that as THI at loading increased from ~19 to 24, dead and total transport losses increased by 3 pigs/trailer (*p* = 0.01, Figure 4 and Figure 5 respectively).

In addition, there were no effects of trailer stocking density on non-ambulatory, dead, or total transport losses (*p* = 0.51, R^2^ = 0.03; *p* = 0.66, R^2^ = 0.07; *p* = 0.68, R^2^ = 0.08; data not presented). The current study reviewed 131 loads and recorded transport losses at 0.05 non-ambulatory, 0.17 dead, and 0.21 total transport losses/trailer. These values are very low. This may explain why the results differ from Fitzgerald *et al*. [10] reviewed 12,333 loads and observed 0.99 non-ambulatory, 0.42 dead, and 1.41 total transport losses pigs/trailer. The authors noted that total transport losses increased with increasing THI and trailer stocking density respectively.

## 4. Conclusions

Stressors during transportation have been shown to be additive [11]. Therefore, reducing or preventing stressors may improve pig well-being [10,20,28]. A variety of factors may influence market weight pig animal based well-being measures and transport losses. The current study did not observe detrimental effects of bedding level in animal based measures or transport losses. It was interesting to note, that increased bedding level did not improve absorption during multiple loads. The authors recommend future pig transport studies to use both THI and trailer stocking density in the statistical model as both had effects on animal based- and transport loss measures. It is extremely important to note that the inference space of this study is relatively small covering only a short time period within a year (during July in Iowa) and a single genetic cross (PIC). Further studies should be conducted to see if these results apply to other geographic regions, seasons and across many commercial genotypes to identify these effects, if any exist.
